# Signal loss due to oligomerization in ELISA analysis of amyloid-beta can be recovered by a novel sample pre-treatment method

**DOI:** 10.1016/j.mex.2015.02.011

**Published:** 2015-02-27

**Authors:** Leen Janssen, Frank Sobott, Peter P. De Deyn, Debby Van Dam

**Affiliations:** a Laboratory of Neurochemistry and Behavior, Institute Born-Bunge, University of Antwerp, Antwerp, Belgium; b Biomolecular & Analytical Mass Spectrometry and Center for Proteomics (CFP-CeProMa), Department of Chemistry, University of Antwerp, Antwerp, Belgium; c Department of Neurology and Memory Clinic, Hospital Network Antwerp (ZNA) Middelheim and Hoge Beuken, Antwerp, Belgium; d University of Groningen, University Medical Center Groningen (UMCG), Department of Neurology and Alzheimer Research Center, Groningen, The Netherlands; e Biobank, Institute Born-Bunge, University of Antwerp, Antwerp, Belgium

**Keywords:** Sample pre-treatment for amyloid-beta ELISA analysis, Alzheimer’s disease, Amyloid-beta, ELISA, Sample pre-treatment, Oligomers, Steric hindrance, AD, Alzheimers disease, Aβ, amyloid-beta, TFA, trifluoroacetic acid, HFIP, hexafluoroisopropanol, FA, formic acid, DMSO, dimethyl sulfoxide, SDS, sodium dodecyl sulphate, WT, wild-type, PMSF, phenylmethylsulfonyl fluoride, SP, soluble proteins, PBS, phosphate-buffered saline

## Abstract

According to the predominant theories, soluble amyloid-beta (Aβ) aggregates are the principal neurotoxic agents in Alzheimer’s disease pathology, making them a popular target for the development of therapeutics and diagnostic markers. One of the most commonly used methods for determining the concentration of Aβ is ELISA. However, ELISA was developed for monomeric proteins and may be ill-suited for detecting aggregates. Therefore, we investigated the effect of aggregation on the ELISA measurement and developed a novel chemical pre-treatment method, designed to disaggregate Aβ peptides, to improve the ELISA measurement of the total Aβ concentration.

Synthetic Aβ40 monomers, Aβ42 oligomers and biological samples from mice and humans were subjected to a chemical pre-treatment protocol with: trifluoroacetic acid (TFA), formic acid (FA) or hexafluoroisopropanol (HFIP) prior to ELISA analysis. In our study we have shown that:

• Aβ oligomerization leads to epitope masking and steric hindrance and results in an underestimation of the total Aβ content with ELISA.
• Chemically pre-treating samples to disaggregate oligomers can (partially) recover the signal loss.
• This novel sample pre-treatment method could provide a more accurate ELISA measurement of the total Aβ concentration in samples with a high oligomer content.

## Graphical abstract

**Figure:**
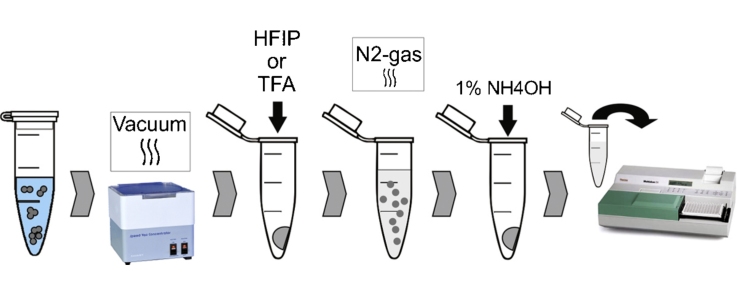

## Method details

**Fig. 1:**
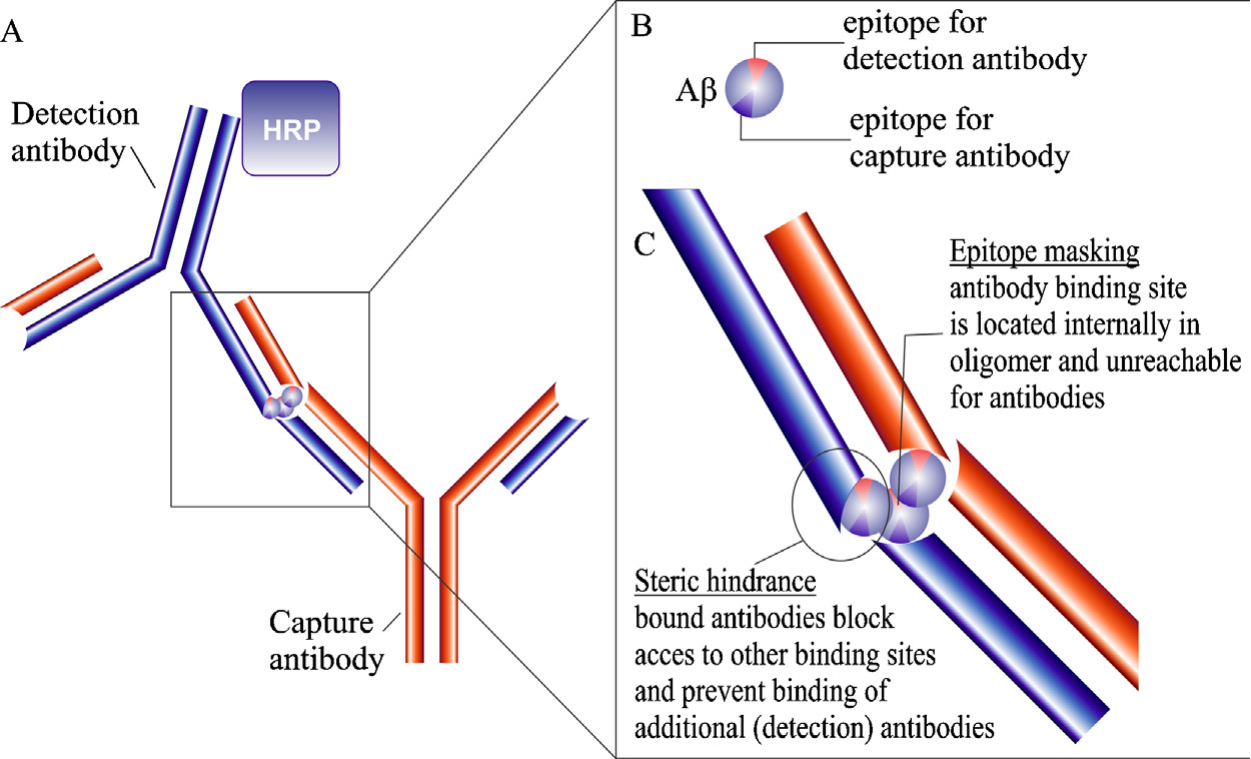
Illustration of antibody binding to Aβ oligomers in ELISA measurement (A). Enlarged representation of the Aβ monomer within the Aβ oligomer (B). Close up of antibody binding illustrating steric hindrance and epitope masking principles (C). Abbreviations: HRP, horseradish peroxidase.

Oligomerization of amyloid-beta (Aβ) can lead to epitope masking and steric hindrance, which can result in an underestimation of the Aβ concentration in ELISA analysis (Fig. 1). We, therefore, decided to develop a chemical pre-treatment protocol to disaggregate Aβ oligomers in samples prior to analysis. Three candidate chemicals with disaggregation properties, namely trifluoroacetic acid (TFA) [1], hexafluoroisopropanol (HFIP) [2] and formic acid (FA) [3] were selected from literature and applied to various samples to evaluate efficacy of the protocol and to identify the most promising option for future research. First, the pre-treatment protocol was tested with synthetic Aβ monomer solutions to assess any unwanted effects. Next, we progressed to solutions of aggregated synthetic Aβ to determine the efficiency of the various treatments. Finally, we examined the compatibility of the protocol with biological samples, i.e., brain extract of the APP23 mouse model for AD and cerebrospinal fluid from human AD patients and control individuals.

### Synthetic Aβ stock solutions

Monomeric Aβ stock solutions were prepared from the human Aβ1-40 standard provided with the Human Amyloid β (1-x) Assay kit (IBL International). In order to obtain structurally homologous and unaggregated peptides for consistent and reproducible aggregation, synthetic Aβ1-42 (AnaSpec) was processed and aliquoted in accordance with the protocol described by Stine et al. in 2003. The aliquots were stored at −80 °C until use.

Preparation of stock solutions of synthetic Aβ oligomers [4]:

• Resuspend Aβ1-42 aliquots to 5 mM in anhydrous dimethyl sulfoxide (DMSO).
• Sonicate for 10 min in bath sonicator.
• Dilute to 100 μM with ice-cold PBS + 0.05% sodium dodecyl sulfate (SDS).
• Allow aggregation for 24 h at 4 °C.
• Dilute to 50 μg/ml and incubated at 4 °C for 2 weeks to allow higher-order aggregation.

Stock solution concentrations were all determined based on the initial Aβ monomer concentration.

### Mouse brain protein extracts

Protein extracts were obtained from brain tissue of 18-month-old wild-type (WT) and heterozygous APP23 mice. All mouse brains were collected with the approval of the ethical committee for animal testing at the university of Antwerp (case number: 2013-61). Hemi-forebrains were stored at −80 °C. By using two extraction buffers in combination with varying degrees of mechanical tissue dissociation, two protein fractions were obtained (protocol adapted from [5]).

## Procedure

• Thaw hemi-forebrains on ice.
• Add 500 μl of ice-cold TNT-buffer [50 mM Tris-base (Thermo Fisher Scientific), 150 mM NaCl, 0.1% Triton X-100, 1 mM phenylmethylsulfonyl fluoride (PMSF), 2 mM 1,10-phenanthroline monohydrate (Merck Millipore), 1× protease inhibitor cocktail (Sigma–Aldrich), pH 7.40].
• Dissociate tissue with a 1-ml syringe (three aspirations without a needle and five aspirations with a 20-gauge needle).
• Dissociated further by pipetting up and down 8–10 times with a 1-ml pipette.
• Centrifuge at 18,000 × *g* for 90 min at 4 °C.
• Collect the supernatant and centrifuge a second time at 18,000 × *g* for 90 min at 4 °C.
• Collect the supernatant of this second centrifugation step and store at −20 °C. This supernatant contains the fraction of soluble proteins (SP).
• Resuspend the pellet of the first centrifugation step in 750 μl RIPA-buffer [50 mM Tris-base (Thermo Fisher Scientific), 150 mM NaCl, 0.5% Triton X-100, 1 mM ethylenediaminetetraacetic acid, 3% SDS, 1% deoxycholate, 1 mM PMSF, 2 mM 1,10-phenanthroline monohydrate (Merck Millipore), 1× protease inhibitor cocktail (Sigma–Aldrich), pH 7.40].
• Vigorously dissociate 20 times with a 1-ml pipette.
• Vortex 20 s.
• Placed on an overhead shaker (Heidolph) for 15 min at 4 °C.
• Centrifuge at 18,000 × *g* for 90 min at 4 °C.
• Collect supernatant and centrifuge a second time at 18,000 × *g* for 90 min at 4 °C.
• Collect the supernatant and store at −20 °C. This supernatant contains the fraction of membrane-bound proteins.

In this particular optimization study, only the SP fraction was used.

### Human CSF samples

Twelve CSF samples from pathologically confirmed AD patients and thirteen control samples with a similar age- and gender-profile were selected from the Biobank facilities of the Institute Born-Bunge (Antwerp, Belgium). Unlike the AD patient samples, control samples were not autopsy confirmed. However, they did not present with central nervous system pathology after neurological work-up and neuropsychological examination revealed no cognitive deficits at the time of CSF sampling. Samples were collected during the clinical work-up of patients in compliance with the Helsinki declaration and with the approval of the ethics committees of the ZNA hospitals and the University of Antwerp. All samples were stored at −80 °C. Three control samples and one AD patient sample had undergone one freeze/thaw cycle prior to analysis. All other samples had never been thawed before. See Supplementary materials for a detailed overview of the study population.

### Sample pre-treatment protocol

• Dry samples in a Savant™ SpeedVac™ concentrator (Thermo Fisher Scientific).
• Reconstitute in phosphate-buffered saline (PBS), TFA, HFIP or FA.
• Place samples in a sonication bath for 15 min.
• Remove TFA and HFIP by drying under a constant stream of nitrogen gas.

or

• Remove FA and PBS by a second run in the concentrator.
• Reconstitute the samples in ultrapure water, PBS or 1%NH_4_OH just prior to analysis.

The samples were also diluted sequentially with each reconstitution step to obtain concentrations within the measurement range of the applied analytical method.

Additional tips:

• To facilitate reconstitution, highly concentrated samples should be diluted a first time prior to starting the protocol and the first drying step.
• The volume for reconstitution should preferably exceed the volume of the sample before drying. Smaller volumes should be avoided at all costs.
• Drying times can vary for different volumes and buffers and should be optimized. While samples should be dried completely, excessive drying should be avoided.

## ELISA measurements

ELISA measurements were carried out using the Human Amyloid β (1-x) Assay kit (IBL International) in accordance with the manufacturer’s instructions. This kit has a detection range between 7.81 and 500 pg/ml and can detect Aβ forms of various lengths, ranging from 28 to 42 amino acids, provided they show no N-terminal modification. Cross reactivity with N-terminally modified Aβ amounts to ≤0.1%.

## SDS-PAGE and Western blotting

Aggregation and disaggregation of standard solutions were evaluated using SDS-PAGE and Western blotting. NuPAGE^®^ LDS sample buffer was added to the samples prior to gel loading. Denaturing, non-reducing SDS-PAGE was performed with the Xcell SureLock mini-cell system (Life Technologies) according to the standard protocol using pre-cast NuPage^®^ 4–12% bis-Tris gels and NuPAGE^®^ MES SDS running buffer (Life Technologies). After electrophoresis, the proteins were blotted to Immobilon^®^-PSQ membrane (Millipore) in the XCell II™ Blot Module (Life Technologies) using the standard manufacturer’s protocol. Aβ was labeled overnight at 4 °C with 6E10-antibody (Covance) and horseradish peroxidase-conjugated anti-mouse antibody (Dako). The blocking buffer consisted of Tween^®^20-Tris buffered saline [10 mM Tris-base (Thermo Fisher Scientific), 200 mM NaCl (Merck Milipore), 0.1% Tween20 (Bio-Rad Laboratories), pH 7.40] and 5% bovine serum albumin 98% grade (Merck Milipore). Protein bands were visualized using the SuperSignal™ West Femto chemiluminescent substrate (Thermo Fisher Scientific). Blots were imaged and analysed with the G:Box imager equipped with Genesnap and Genetools software (Syngene).

## Statistical analysis

All statistical tests were performed with the level of probability set at 95% using IBM SPSS statistics 22.0.0 software (IBM). Low inter-sample variability was expected in analyses of standard stock solutions. Extreme outliers (values exceed more than three times the interquartile range) were suspected to be the result of procedural aberrations and therefore excluded from the dataset. The various treatment groups of the standard solution were compared using a one-way ANOVA with a Bonferroni post-hoc test. The genotype groups of the brain extract samples were compared for each treatment with the independent-samples Mann–Whitney *U* test. A paired statistical analysis, using the related samples Wilcoxon signed rank test, was performed to compare the measurements of untreated brain extracts to the measurement of the sample after a given treatment. The results of the analysis of the human CSF samples were analysed by a two-way repeated measures ANOVA with a Bonferonni post hoc test for the comparison between treatment groups.

## Method validation using monomeric Aβ stock solution

**Fig. 2:**
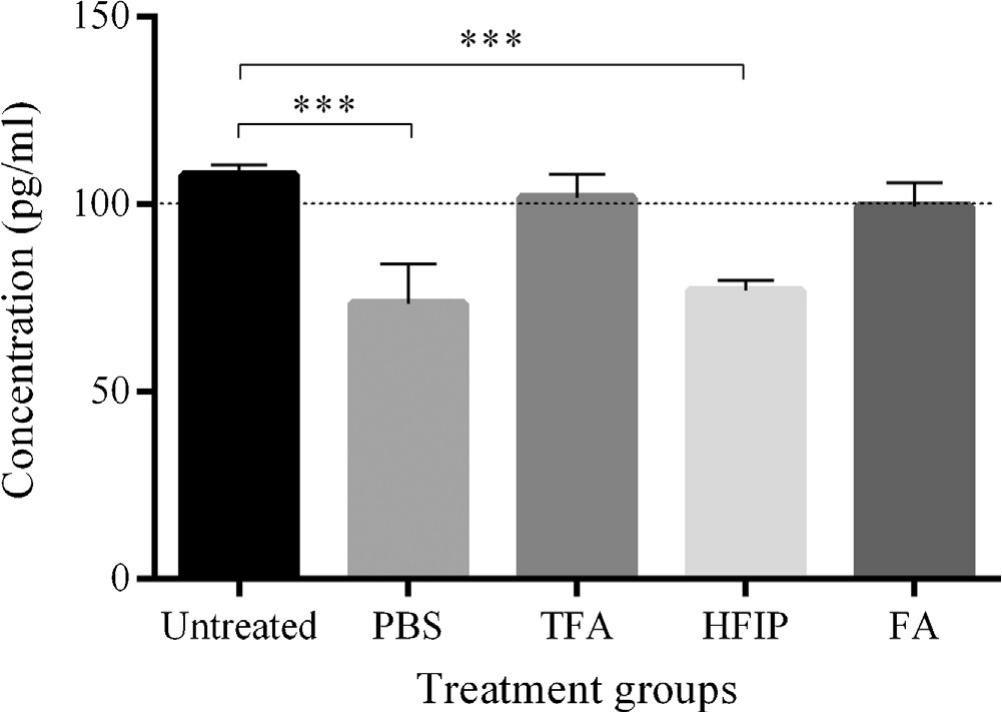
Results of ELISA measurements of Aβ_40_ monomer standard solution. Comparison of means (±SD) between the untreated (*n* = 7), PBS (*n* = 8), TFA (*n* = 8), HFIP (*n* = 8) and FA group (*n* = 7). Asterisks represent significant differences between the untreated group and treatment groups (post-hoc Bonferroni test; ***p < 0.001). The dashed line represents the standard’s theoretical concentration. Abbreviations: FA, formic acid; HFIP, hexafluoroisopropanol; PBS, phosphate-buffered saline; TFA, trifluoroacetic acid.

Initially, we investigated whether the chemical treatments had any unwanted effects on the ELISA measurement of Aβ by subjecting our standard solution of synthetic Aβ 1-40 monomers (0.1 ng/ml) to the various treatments. ELISA results indicated significant, unwanted effects in some treatment groups (*F*_4,37_ = 42.813; *p* < 0.0001; Fig. 2). Post-hoc Bonferroni tests between the individual groups showed there was no significant difference between measurements of untreated standard and TFA-treated (*p* = 0.884) or FA-treated (*p* = 0.231) standard. On average, these three groups showed minimal deviation (<10%) from the theoretical standard concentration, demonstrating no adverse effect of these sample pre-treatments on sample recovery. The control group, treated with PBS, and the HFIP-treated group did appear to experience a significant reduction in signal due to the procedure, when compared to the untreated group (both *p* < 0.0001; Fig. 2). The fact that the PBS control group was affected and that there was no real difference between the PBS group and HFIP group (*p* = 1.000), seems to indicate that the observed effect is caused by the process of drying and reconstituting the samples, rather than by the chemical itself.

## Method validation using oligomeric Aβ stock solution

**Fig. 3:**
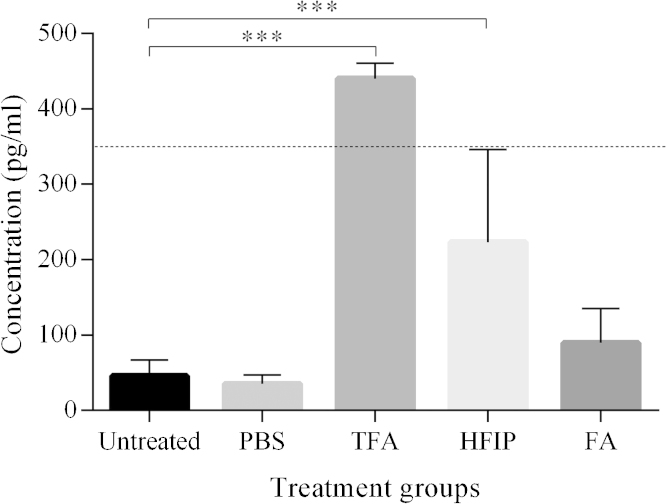
Results of ELISA measurements of Aβ_42_ oligomer standard solution. Comparison of means (±SD) between the untreated (*n* = 17), PBS (*n* = 16), TFA (*n* = 7), HFIP (*n* = 10) and FA group (*n* = 8). Asterisks represent significant differences between the untreated group and treatment groups (post-hoc Bonferroni test; ***p < 0.001). The dashed line represents the standard’s theoretical concentration. Abbreviations: FA, formic acid; HFIP, hexafluoroisopropanol; PBS, phosphate-buffered saline; TFA, trifluoroacetic acid.

**Fig. 4:**
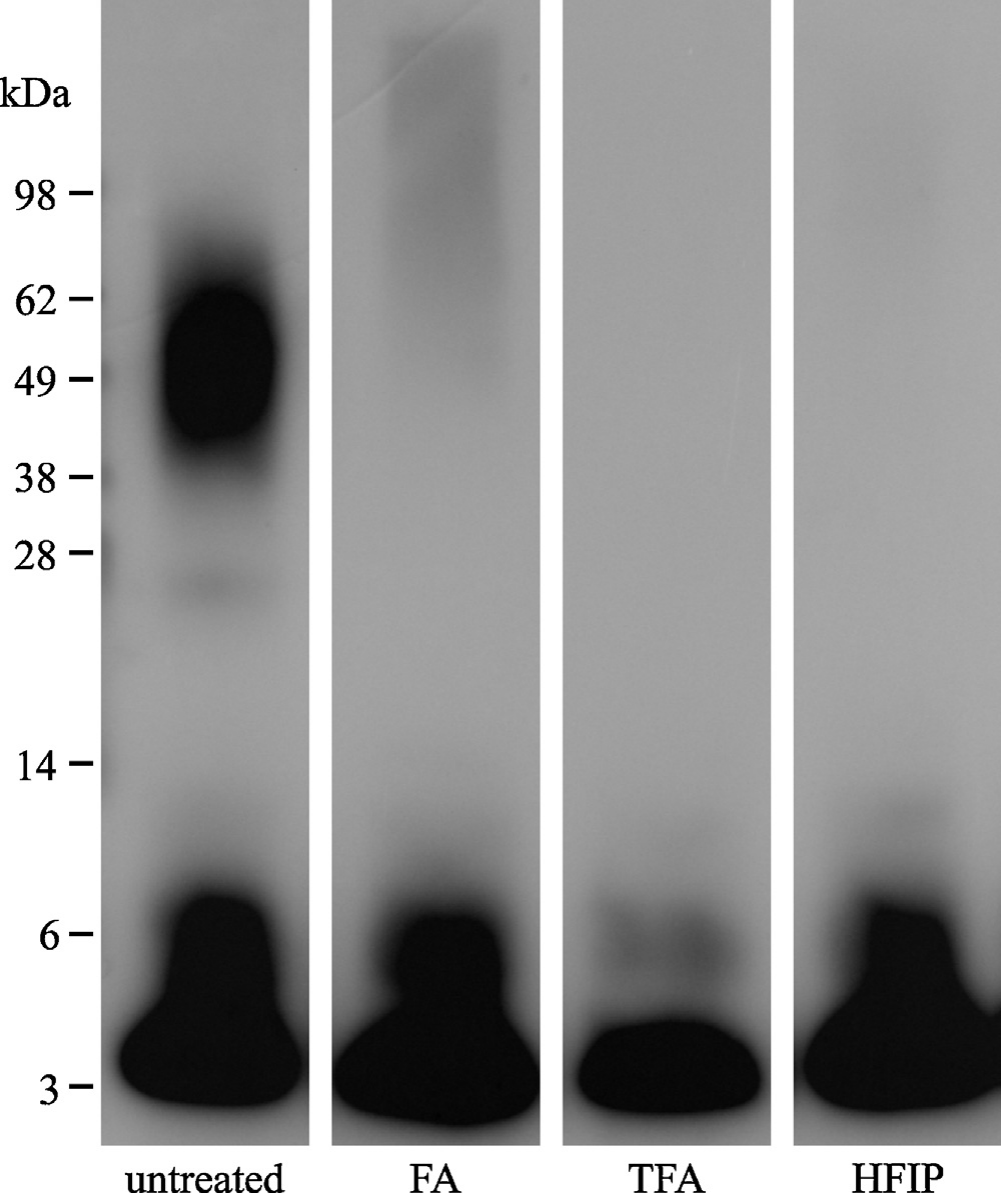
Western blot results after SDS-PAGE of the Aβ_42_ oligomer standard solution without treatment and after treatment with FA, TFA, or HFIP. Abbreviations: FA, formic acid; HFIP, hexafluoroisopropanol; TFA, trifluoroacetic acid. (Lanes were cropped from one blot, for the full image, see Supplementary materials).

Next, we investigated the effects of the various chemical treatments on a standard solution of synthetic Aβ1-42 oligomers, which were serially diluted to a concentration of approximately 0.350 ng/ml (concentration based on the initial Aβ monomer concentration at the start of the aggregation procedure) prior to analysis. ELISA results showed no significant difference between the untreated and the PBS-treated standard (*p* = 1.000; Fig. 3). However, the measured Aβ levels for both groups averaged 10–15% of the theoretical standard concentration, demonstrating the impact of oligomers on ELISA measurements. While the FA treatment did result in a slight increase in the Aβ signal, the signal did not differ significantly from the untreated group (*p* = 0.715) and still only reached about 25% of the theoretical value. Treatment with TFA or HFIP did yield a significant increase in signal compared to the untreated group (both *p* < 0.0001). Despite the fact that HFIP treatment showed a decrease in signal in the tests with the monomer standard, the mean value of the HFIP-treated group actually came close to 65% of the theoretical concentration. The TFA group even exceeded the theoretical value. However, given that this theoretical value was calculated based on the serial dilution of the original stock solution and that ELISA tests are not 100% accurate, some deviation can be expected. Western blotting confirmed disaggregation by all three treatments, although some low *n*-oligomers could still be observed (Fig. 4). It is unclear, however, whether these oligomers were unaffected by the treatment, re-formed during the SDS-PAGE and Western blotting procedure or a combination of both. As the use of SDS has been associated with the induction of Aβ oligomerization [6], it cannot be excluded that the observed low *n*-oligomers are an artefact caused by the SDS in the procedure. The TFA treatment did show the least amount of these low *n*-oligomers and the ELISA results seem to indicate a (near) complete disaggregation after TFA. TFA would thus appear to be the most efficient treatment in these experiments.

## Method validation using biological samples

**Fig. 5:**
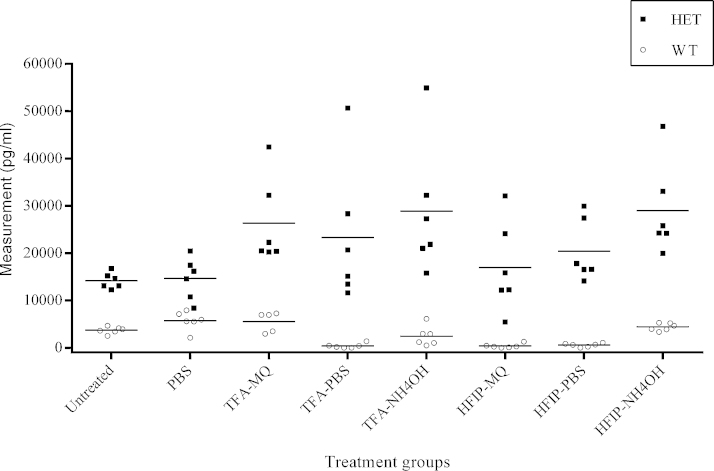
ELISA results of brain extracts. Comparison of heterozygote (black squares) and wild-type (open circle) samples without treatment and after PBS, TFA–MQ, TFA–PBS, TFA–1%NH_4_OH, HFIP–MQ, HFIP–PBS or HFIP–1%NH_4_OH treatment. Horizontal lines represent the mean for each genotype per treatment. Abbreviations: FA, formic acid; HFIP, hexafluoroisopropanol; MQ, ultrapure water; PBS, phosphate-buffered saline; TFA, trifluoroacetic acid.

**Fig. 6:**
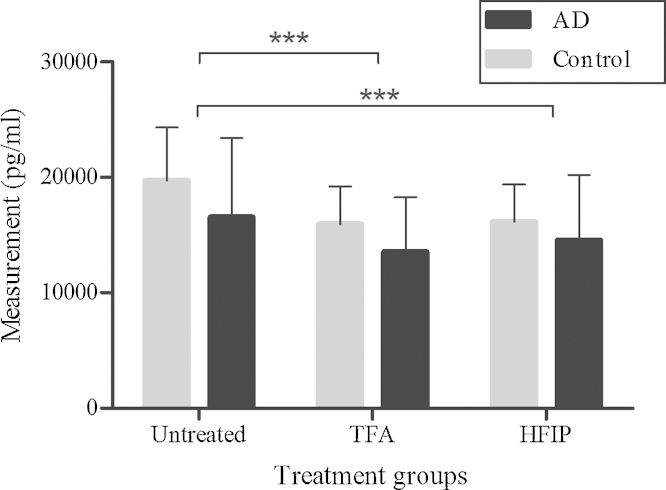
Elisa results of human CSF samples. Comparison of means (±SD) between AD patient (*n* = 12) and control (*n* = 13) samples without treatment and after treatment with TFA or HFIP. All treated samples were reconstituted in 1%NH_4_OH. Asterisks represent significant differences between the untreated group and treatment groups (post-hoc Bonferroni test; ***p < 0.001). Abbreviations: AD, Alzheimer’s disease; HFIP, hexafluoroisopropanol; TFA, trifluoroacetic acid.

Finally, we explored the effect of sample pre-treatment on biological samples, i.e., protein extracts from brain tissue of APP23 mice (Fig. 5) and human CSF samples (Fig. 6). Since the FA treatment failed to produce a significant effect in the ELISA measurements of the oligomer standard, it was not included in these further experiments. Due to the more complex matrix of the brain extracts and the fact that we previously diagnosed a possible problem with reconstitution after HFIP treatment, we did include three reconstitution solutions for comparison: ultrapure water, PBS and a 1% NH_4_OH solution. Since WT mice do not express human Aβ, all treatment groups showed a clear difference in Aβ measurements between WT and heterozygous mice (*p* < 0.005). The more important question is of course whether the pre-treatments resulted in an increase of the ELISA signal in samples of heterozygous mice similar to the increase observed with the oligomer standard. When comparing the results of the heterozygote samples with and without treatment, we found no significant differences between the untreated samples and the PBS-treated (*p* = 0.917), the TFA–PBS-treated (*p* = 0.249) and the HFIP–ultrapure water-treated samples (*p* = 0.600). The TFA–ultrapure water treatment group, the TFA–1%NH_4_OH group, the HFIP–PBS group and the HFIP–1%NH_4_OH group all exhibited a significant increase in signal (*p* < 0.05). Ideally, the WT samples are unaffected by the treatment. In reality, however, the signal of WT samples was lowered significantly in the TFA–PBS, the HFIP–ultrapure water and the HFIP–PBS group (*p* < 0.05). On the other hand, in the PBS-treated group a slight increase was observed (*p* = 0.046). The other treatment groups did not display a significant difference between untreated and treated WT samples. As 1%NH_4_OH appeared to be the most efficient reconstitution buffer for both TFA and HFIP-treated brain extracts, we selected this reconstitution buffer for the experiments with human CSF. Contrary to the results from the brain extracts, treatment of the CSF samples resulted in a decrease in the ELISA signal for both TFA-treated and HFIP-treated samples when compared to the untreated samples (*p* < 0.001, Fig. 6). No significant difference could be observed between the control samples and the AD samples (*p* = 0.217) and the effect of the various treatments was also similar for both groups (*p* = 0.230). Treatment of the brain extract samples resulted in an increased scatter of the data. Whether this simply reflects the biological variability in Aβ aggregation or is an issue of consistency/reproducibility of the treatment procedure, remains to be investigated. The human CSF samples, however, did not show an increased scatter after treatment. Before applying the protocol, it should be extensively validated for reproducibility and proper controls should be included in any experiment.

## Additional information

### Background

Ever since its first description by Dr. Alois Alzheimer in 1906, Alzheimer’s disease (AD) has been associated with two distinctive histopathological lesions: amyloid plaques of aggregated Aβ and neurofibrillary tangles of hyperphosphorylated tau. Subsequent Alzheimer research has, therefore, mainly focused on elucidating the relationship between these proteins and the observed symptomatology in the hope of identifying diagnostic markers and possible therapeutic targets. This research uncovered significant genetic and biochemical evidence (for reviews [7], [8], [9], [10]), which contributed to the development of the amyloid cascade hypothesis. According to this hypothesis, the Aβ peptide plays a central role in AD pathology [11], rendering Aβ the focal point of many research endeavors. It became apparent, however, that the fibrillar Aβ in plaques correlated poorly with the cognitive impairment observed in AD patients [12], [13]. Soluble, non-fibrillar Aβ aggregates, however, correlate strongly with disease severity and synaptic alterations [14], [15], [16]. These discoveries led to a more refined version of the amyloid cascade hypothesis in which soluble Aβ aggregates are cast as the principal neurotoxic agent in AD pathology [17]. To this day, this hypothesis is still the most widely accepted theory and Aβ remains a popular target for the development of therapeutics and diagnostic markers. In fact, several imaging and biochemical methods have already been developed to determine Aβ levels for diagnostic purposes [18], [19], [20], [21], [22], [23]. One of the most commonly used tools for the determination of Aβ concentrations is an immunoassay. During recent revisions of the diagnostic criteria for AD, ELISA-based analyses were even included as a possible supportive measure in the diagnosis of AD [24], [25]. ELISA, however, was designed for the detection of monomeric proteins. Unfortunately, Aβ has a tendency to aggregate and, as mentioned earlier, these aggregates are considered to be crucial to the pathology. The capacity of immunoassays to properly detect these aggregates and accurately determine the total Aβ concentration has been called into question [26], [27]. ELISA measurements are based on the principle that each target protein will bind a single enzyme-conjugated antibody, resulting in a measurable signal that is proportional to the quantity of protein in the sample. Upon aggregation, however, the hydrophobic C-terminus of Aβ gets internalized in the core of the aggregate [28], giving rise to epitope masking, i.e., the antibody binding site becomes inaccessible for the antibody (Fig. 1). In addition, given the relatively small size of Aβ (∼4–5 kDa) and the large size of the ELISA antibodies (∼150 kDa), steric hindrance could also inhibit the binding of multiple detection antibodies in close proximity of each other (Fig. 1). Spatial limitations might prevent some of the Aβ peptides in an aggregate from binding to a detection antibody and contributing to the measured concentration. Together, these two effects make it impossible to determine how many antibodies, if any, a given aggregate will bind. The obtained signal will, therefore, not accurately represent the total Aβ content and lead to an underestimation of the actual concentration. Due to these inherent problems with the traditional ELISA and the important role of soluble Aβ aggregates in AD pathology, several research groups have been developing new immunoassays to specifically target Aβ aggregates. Some of these groups have chosen to adapt the traditional ELISA by using aggregate-specific antibodies [29], [30], [31], [32], or by using a single monoclonal antibody for both antigen capture and detection [29], [32], [33], [34], [35], [36], [37]. Others groups apply techniques like a bio-barcode assay [38], surface-fluorescence intensity distribution analysis [39] or fluorescence resonance energy transfer in combination with flow cytometry [40]. With the exception of the assays using aggregate-specific antibodies, all these methods still rely on the binding of multiple antibodies and are vulnerable to the effects of epitope masking and steric hindrance. The aggregate-specific assays are entirely reliant on the selection of an appropriate conformational antibody and run the risk of merely detecting a subset of oligomers with that specific conformation. Finally, none of these tests provides information on the total Aβ level. Therefore, instead of trying to modify immunoassays to detect whole aggregates, another approach might be to disaggregate Aβ in samples prior to analysis. After all, the ELISA technique was designed to provide quantitative and not structural information. As this approach will result in the loss of aggregate structures in the samples, information about the aggregate concentration and the degree of aggregation will be lost. Where aggregate specific tests provide information on aggregate concentrations, this method measures the total concentration of Aβ monomers. However, it might be possible to deduce some information about the degree of aggregation in samples by calculating the before/after treatment ratio.

## Conclusions

Overall, our study has shown that the presence of oligomers in samples can seriously affect the accuracy of ELISA measurements of the total Aβ content. The validation experiment with the oligomeric stock solution clearly shows a decrease in signal and the resulting underestimation of the Aβ content. In samples with a significant oligomer content, like the oligomer stock solution and the brain extracts, both TFA and HFIP treatment appeared capable of disaggregating oligomers and recovering the lost signal. In samples with a low oligomer content, like the monomeric stock solution or the CSF samples [31], [35], [36], [37], the pre-treatment method did not induce an increase in signal, which is to be expected. On the contrary, some samples actually showed a loss in signal, most likely due to incomplete protein recovery upon reconstitution after drying. As the effect of oligomers on the accuracy of the ELISA measurements will be negligible in samples with low oligomer content, pre-treatment of these samples is inadvisable. The 1% NH_4_OH solution appeared to be the best option for reconstitution in our experiments, but some additional optimization might be necessary depending on the samples and analytical method used.
