# Supplementary material for: Signal loss due to oligomerization in ELISA analysis of amyloid-beta can be recovered by a novel sample pre-treatment method
Source: MethodsX. 2015 Feb 27;2:112–23. doi: 10.1016/j.mex.2015.02.011 (PMC4487349; doi:10.1016/j.mex.2015.02.011)
Supplement: Supplementary file 1 [file mmc1.pdf]

Supporting information

Dataset ELISA measurements of monomeric Aβ_{1-40} standard solution

Untreated	PBS	TFA	HFIP	FA
106,036	64,551	106,657	80,925	94,608
102,917	67,277	105,757	77,348	97,729
107,836	58,282	96,895	73,446	94,119
130,206*	70,286	101,820	78,296	96,484
110,907	74,556	97,640	77,776	19,912*
108,786	89,055	94,408	73,506	98,639
106,606	87,587	97,471	74,418	101,060
110,775	75,931	113,063	79,193	112,560

Raw data from ELISA measurements of the synthetic monomeric Aβ standard. Samples are unpaired between treatment groups. * indicates extreme outliers with values exceeding more than three times the interquartile range, which were excluded from further the statistical analysis.

Abbreviations: FA, formic acid; HFIP, hexafluoroisopropanol; PBS, phosphate-buffered saline; TFA, trifluoroacetic acid.

Dataset ELISA measurements of oligomeric Aβ_{1-42} standard solution

Untreated	PBS	TFA	HFIP	FA
47,808	43,975	462,003	241,586	70,241
46,778	46,588	438,532	270,419	58,793
99,584	52,368	418,167	268,153	46,845
84,26	39,529	416,321	407,664	77,725
40,288	38,182	461,237	271,672	86,316
42,704	31,417	457,441	202,602	134
32,115	35,187	429,525	160,883	181,598
34,59	28,86	86,704*	43,69	60,723
39,149	28,857	23,242*	349,336	
25,899	20,964		16,794	
20,686	61,491			
50,102	16,09			
43,429	23,967			
64,473	20,882			
23,383	40,513			
28,49	32,845			
56,457	183,000*			

Raw data from ELISA measurements of the synthetic Aβ oligomer standard. Samples are unpaired between treatment groups. * indicates extreme outliers with values exceeding more than three times the interquartile range, which were excluded from further the statistical analysis.

Abbreviations: FA, formic acid; HFIP, hexafluoroisopropanol; PBS, phosphate-buffered saline; TFA, trifluoroacetic acid.

Full western blotting image of Aβ oligomer standard with and without treatment

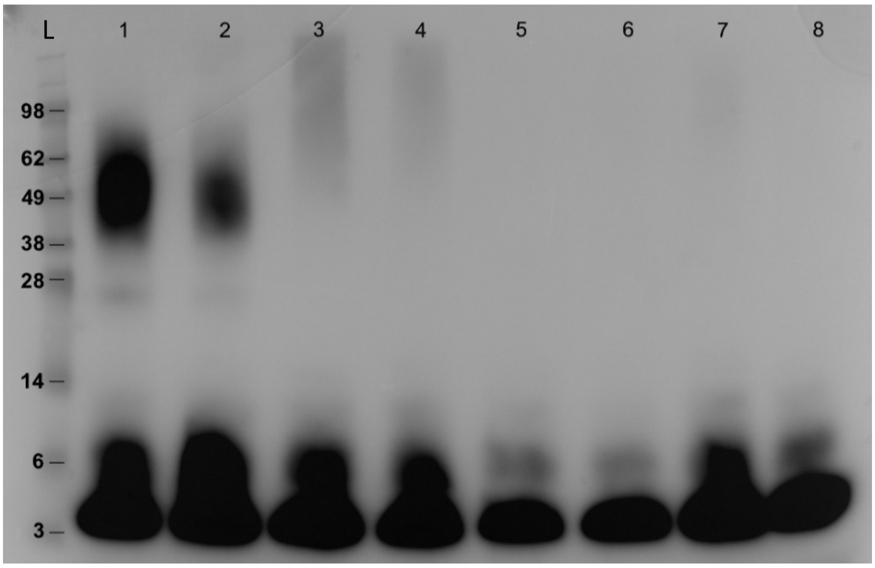

L: protein marker ladder (kDa)
Lane 1-2: untreated A$\beta_{1\text{-}42}$ oligomer standard
Lane 3-4: Formic acid-treated A$\beta_{1\text{-}42}$ oligomer standard
Lane 5-6: Trifluoroacetic acid-treated A$\beta_{1\text{-}42}$ oligomer standard
Lane 7-8: Hexafluoroisopropanol-treated A$\beta_{1\text{-}42}$ oligomer standard

Dataset ELISA measurements of mouse brain extracts

Mouse nr.	Untreated	PBS	TFA-MQ	TFA-PBS	TFA-NH4OH	HFIP-MQ	HFIP-PBS	HFIP-NH4OH
WT 1	2506,51	2091,7	20758,50*	1362,05	6083,27	261,295	817,472	5242,3
WT 2	3899,38	7092,42	6944,72	0	2927,4	0	0	4652,04
WT 3	3406,9	7880,2	6935,14	0	2879,59	62,12	644,76	5178,73
WT 4	3595,04	5591,92	7231,92	143,04	1173,47	1243,69	1051,8	3872,1
WT 5	4141,13	5535,17	3473,44	423,06	481,43	438,31	590,9	3960,23
WT 6	4646,88	5911,28	2951,97	384,91	1019,02	193,18	216,92	3350,67
HET 1	12241,4	8394,81	32193	20628,9	27292,9	15797	16537,7	25793,3
HET 2	13081	10747,4	42398,3	50624,2	54927,5	32052,3	29931,1	46775,1
HET 3	16748,5	20424,7	20205,3	28284,5	32187,9	24074,2	27421,5	33032,3
HET 4	14611,4	14560,8	22282,4	15095,4	20980,7	12146,4	17769,3	24250,9
HET 5	13069,5	16180,3	20388,8	11578,3	15763,5	5439,06	14080,1	19955,2
HET 6	15201	17452,8	20455,2	13452,9	21849,8	12251,2	16501,3	24161,7

Raw data from ELISA measurements of protein extracts from the brain of 18-month-old APP23-mice and wild type littermate controls. Samples are paired between treatment groups. * indicates extreme outliers with values exceeding more than three times the interquartile range, which were excluded from further the statistical analysis. “0” indicates values that fell below the lower limit of detection of the test. Abbreviations: FA, formic acid; HET, heterozygous APP23-mouse; HFIP, hexafluoroisopropanol; MQ, ultrapure water; PBS, phosphate-buffered saline; TFA, trifluoroacetic acid; WT, wild type control.

Summary of human study population data

	AD	Control
Total Number (Male/Female)	12 (7/5)	13 (8/5)
Mean Age (± SD)	74,4 ± 10,2	73,5 ± 11,3
Mean ELISA measurement untreated (pg/ml ± SD)	16543 ± 6835	19692 ± 4592
Mean ELISA measurement TFA treated (pg/ml ± SD)	13554 ± 4693	15922 ± 3294
Mean ELISA measurement HFIP treated(pg/ml ± SD)	14555 ± 5642	16143 ± 3196

Total number of male and female patients included in the experimental groups and the average age (at sample collection) and ELISA measurements for each group. Abbreviations: AD, Alzheimer's disease; HFIP, hexafluoroisopropanol;SD, standard deviation; TFA, trifluoroacetic acid.

Dataset ELISA measurements of human CSF samples

Patientn°	Gender	Age	Freeze/thaw cycli	Elisa measurements (pg/ml)		
				Untreated	TFA	HFIP
AD 1	M	87	0	10685,4	10017,6	10552,3
AD 2	M	73	0	13631,7	11540,7	10475,6
AD 3	F	66	0	27216,8	16289,5	19694,2
AD 4	F	83	0	22021,3	16267,1	18172,1
AD 5	F	86	0	26558,3	22499,0	25624,2
AD 6	M	70	0	16700,6	15538,7	15367,6
AD 7	M	83	1	11182,9	12887,9	11112,1
AD 8	M	81	0	14715,9	12081,4	13856,7
AD 9	M	60	0	5553,8	4277,6	5324,5
AD 10	F	72	0	14855,4	11751,3	12469,0
AD 11	M	56	0	23549,8	18759,4	20939,8
AD 12	F	75	0	11842,1	10740,9	11070,3
Control 1	M	72	0	25247,2	18150,8	20556,9
Control 2	M	79	0	16825,1	14656,2	14129,1
Control 3	F	88	0	26158,3	18536,8	18600,6
Control 4	M	78	0	25244,4	21311,7	18096,5
Control 5	M	70	1	22171,1	17210,8	17339,6
Control 6	F	81	0	19085,1	15632,8	14684,9
Control 7	F	74	0	16350,1	13338,9	14293,8
Control 8	F	50	0	13415,4	11382,9	12635,4
Control 9	M	50	0	21201,9	14668,4	18969,6
Control 10	M	82	0	22601,5	20900,9	19917,8
Control 11	M	76	0	20128,6	17132,8	16978,8
Control 12	M	83	1	12463,2	11484,8	9624,9
Control 13	M	73	1	15102,7	12571,9	14032,2

Raw data from ELISA measurements of human CSF samples of AD patients and controls including gender and age at sample collection of each test subject, as well as the number of freeze thaw cycli of the samples prior to testing. Samples are paired between treatment groups. Abbreviations: AD, Alzheimer's disease; HFIP, hexafluoroisopropanol;SD, standard deviation; TFA, trifluoroacetic acid.
